# Effects of Plant Density and Fertilization on Agronomic Traits and Yield of Flax (*Linum usitatissimum* L.)

**DOI:** 10.3390/plants14182891

**Published:** 2025-09-18

**Authors:** Panteleimon Stavropoulos, Antonios Mavroeidis, Antigolena Folina, Ioannis Roussis, Stavroula Kallergi, Stella Karydogianni, George Papadopoulos, Ioanna Kakabouki

**Affiliations:** Laboratory of Agronomy, Department of Crop Science, Agricultural University of Athens, 11855 Athens, Greece; stavropoulosp@aua.gr (P.S.); antoniosmauroeidis@gmail.com (A.M.); folinanti@gmail.com (A.F.); roussis@aua.gr (I.R.); alinakallergi1@gmail.com (S.K.); karydogianni@aua.gr (S.K.); gpapadopoulos@aua.gr (G.P.)

**Keywords:** plant density, flax (*Linum usitatissimum* L.), oil content, yield, harvest index, Mediterranean Basin

## Abstract

Flax (*Linum usitatissimum* L.) is a retrovative crop with highly nutritious seeds. The study aimed to assess the effect of plant density and type of fertilizer on crop growth and productivity. A three-year experiment was conducted at the Agricultural University of Athens, focusing on two densities (D1 and D2) and four fertilizers (urea with inhibitors (I), organic (O), urea (U) and control (C)). Measurements included plant height, dry weight, number of capsules, number of seeds per capsule, Thousand Seeds Weight (TSW), yield, Harvest Index (HI), seed oil content and oil yield. Fertilization increased plant height (14.6–15.2%), dry weight (34.1%) and number of capsules (24.9%). D2 improved biomass production by 52.6%, while D1 increased the number of capsules by 16.4%. TSW increased by 6.7 and 23.9% at D2 and fertilized treatments, respectively. Yield, HI and oil yield were affected by the interaction of density and fertilization, while seed oil content was affected only by fertilization. D2I increased yield by 67.9% compared to D2C, while oil yield increased over twofold. Overall, flax represents a promising alternative crop against climate change in the Mediterranean Basin.

## 1. Introduction

Climate crisis and its immediate effect on agriculture [1,2,3,4] have highlighted the potential of alternative crops [5,6] as an adaptation strategy to the rising temperatures, drought and other extreme weather phenomena [7,8,9]. Flax (*Linum usitatissimum* L.) has been described as a retrovative crop for the Mediterranean [6,10]. It is a member of the Linaceae family, while the Latin word “*usitatissimum*” describes the many different uses it has [11,12]. The main uses of the crop are high quality fiber production, known as linen [13,14,15] and seed production, either for consumption as whole seeds [16,17,18,19] or for the extraction of highly nutritious oil [20,21,22,23,24]. The interest in flax seeds consumption is rising [25,26]. The main reason is their high nutritional value. They are rich in alpha-linolenic acid (ALA) [27,28], while the seed protein content is about 10% [29]. Linseed is mainly cultivated in Russia, Kazakstan, Canada, China and the USA [30,31]. In recent years, the interest in flax cultivation in the Mediterranean Region has been rising [32]. Many studies have focused on the sowing and harvest time, irrigation, tillage and fertilization rates [32,33,34,35].

Studies suggest that fertilization and plant density are two major factors influencing flax performance [36]. Sowing densities vary depending on whether flax is cultivated for fiber production (110–140 kg ha^−1^) or seed and oil production (50–60 kg ha^−1^) [37]. Higher densities can lead to the improvement of straw, fiber and seed yield [38,39,40], while lower densities result in the growth of more branches [41], a negative trait for fiber production [38]. Similarly, the use of nitrogen (N) fertilizers can improve crops’ growth and yield [42,43,44]; however, the literature suggests a wide range of N fertilization rates (20–150 kg N ha^−1^) as optimal [45]. Urea is the most common fertilizer [46,47,48], yet the use of inhibited fertilizers has been proven as a viable, less environmental harming and a more efficient alternative to urea [46,47,48,49,50,51,52,53,54,55,56]. This type of fertilizer delays the release of urea in the ground. As a result, losses are lower while the efficiency of the fertilizer is increased [57]. The urease inhibitor thiophosphorictriamide (NBPT) and the nitrification inhibitor dicyandiamide (DCD) are the most common inhibitors. They focus on the reduction in the formation NH_4_^+^ and NO_3_^−^ [58]. Additionally, the use of organic fertilization also has a positive impact on flax crops. Manure [59,60,61,62,63], Plant Growth-Promoting Microorganisms (PGPM) [64,65,66] and other soil amendments can increase seed yield. Among these amendments tomato pomace, a sub-product of the processing of industrial tomatoes [67], has been suggested to improve the agronomic traits and the yield of several crops when combined with other organic fertilizers (manure, compost) [68,69].

Notably, and despite the rising popularity that flax is gaining in some Mediterranean countries, studies conducted in the region have not thoroughly assessed the effect of plant density, fertilization rates, fertilization type and their interaction remain rather limited in the region. According to Kakabouki et al. [35] flax fertilization requirements are estimated at 30–60 kg N ha^−1^, yet in their study they did not evaluate the efficiency of fertilization in different plant densities, nor did they study the effects of different fertilizer types on the crop. Moreover, research on the application of organic fertilizers and soil amendments in flax in the Mediterranean, and their interaction with different plant densities is rather limited. The aim of the present study is to assess the effect of two different plant densities and three different fertilization types in flax, under Mediterranean conditions.

## 2. Results

### 2.1. Agronomic Characteristics

**Figure 1:**
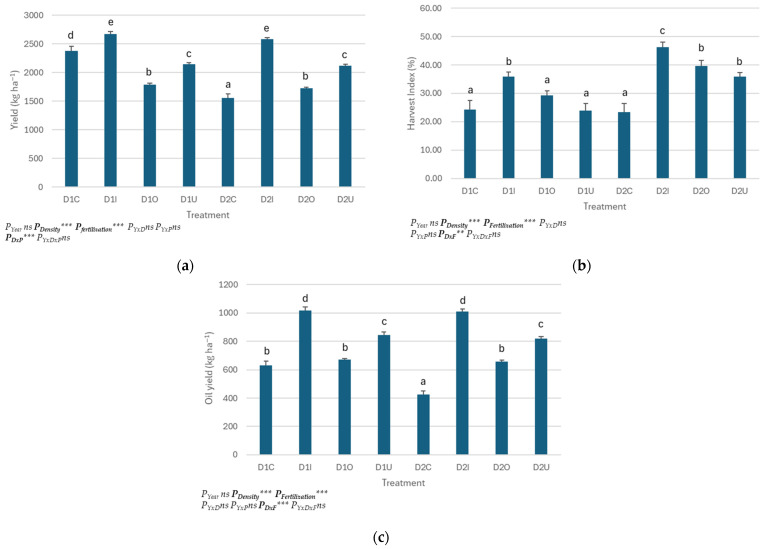
Effect of plants’ density and type of fertilization on flax: (**a**) yield (**b**) Harvest Index (HI) (**c**) oil yield. Values represent the three-year mean (2023–2025). Different letters within the same column indicate statistically significant differences at *p* = 0.05 significance level. The symbol “ns” indicates non statistically significant differences, while the symbols “**” and “***” indicates significance at the *p* < 0.01 and *p* < 0.001 levels, respectively.

All data presented in the tables (Table 1, Table 2, Table 3 and Table 4) and in Figure 1 correspond to mean values calculated across the three-year experimental period (2023–2025). Overall, based on the results of the present study, both fertilization and plant density significantly affected the performance of flax. In particular, the application of I reported the highest yields, number of capsules, and TSW. On the contrary, plant density affected only the number of capsules and the TSW, particularly in D2 where the best results were reported. According to the statistical analysis, fertilization significantly affected the agronomic characteristics of the crop, as presented in Table 1. Plant height was statistically significantly higher in fertilized treatments than the control (unfertilized). The use of urea had a higher impact on plant height, increasing it by 15.2%, compared to the control, while this increase was 14.6% by using the urea +UI +NI. A smaller increase in the same characteristic was also observed by using organic fertilizer (10.5%). The dry weight was also affected by the type of fertilizer. Statistically significant differences were observed at both densities, for the dry weight, where the use of fertilizer increased it by 34.6, 22.3 and 45.6% for I, O and U, respectively, compared to the control (Table 1). The number of capsules was statistically significantly higher (in a mean value of 22.6%) in all of the fertilized plots compared to the control. The number of capsules increased by 27.9, 16.9 and 23.1%, for I, O and U, respectively (Table 1).

Following fertilization, the dry weight and the number of capsules were also affected by density, as shown in Table 2. Dry biomass was higher in D2. Lower density reported a 65.3% increase in the plant’s dry weight. The number of capsules was significantly affected by density (Table 2). The decrease in plants’ density caused a 16.8% increase in the number of capsules per plant.

### 2.2. Yield and Yield Components

The number of seeds per capsule was not significantly affected by any of the factors. Both year, density and fertilization did not statistically significantly influence the number of seeds per capsule (Table 3). The TSW was significantly affected by both density and fertilization. As presented in Table 2, the control reported a 0.2% higher TSW in D2. Fertilized treatments had no significant differences between densities. In D1, C reported the lowest TSW. An increase of 20.2% reported in the use of U, compared to C, while this increase was 28.7 and 40.9%, for O and I, respectively. In D2, the highest TSW was reported in I, with an increase of 27.3%, followed by O (19.4%) and U (8.9%), compared to C. No significant differences were observed in O and I in D2, while in D1, O did not report any statistical differences from both U and I (Table 3).

Crop yield was influenced by the interaction of density and fertilization (P_D×F_ < 0.001). As presented in Figure 1, higher yields were reported at higher densities. The use of fertilizers also increased crop yield. The highest values were reported at the D1I and D2I treatments. No significant differences were observed between these treatments for all the years. The use of urea + UI + NI increased yield by 33.3%, followed by urea with an increase of 8.33%. Organic fertilizer reported the lowest yields for both D1 and D2. Yields in D1 were higher than those in D2. The decrease in plant density caused a 11.3% decrease in the crop’s yield. The combination of high density and urea with inhibitors increased yield by 12.22%, while this increase was 65.5% at the lower density.

Harvest Index (HI) was significantly statistically affected by the interaction of density and fertilization. Urea + UI + NI at D2 reported the highest values of HI (Figure 1). The combination of low density and fertilization with inhibitors almost doubled HI. The addition of fertilizer increased HI by 45 and 26% for organic fertilizer and urea, respectively. Lower values were reported at the control in both D1 and D2, with no significant differences between these two treatments.

The seed oil content was affected by fertilization (Table 4). The use of fertilizers increased oil content by 42.8%, compared to the unfertilized treatment. No statistically significant differences were observed between the fertilized treatments; although, seed oil content was 43.5, 40.2 and 44.6% higher than the control, at urea with inhibitors, organic and urea, respectively. Oil yield was affected by the interaction of plants’ density and fertilization (Figure 1). The highest oil yield was reported at D1I and D2I, where it was doubled, compared to the control. Lower density increased oil yield by 10.2%, while the use of fertilizers increased it by 58.2%. Inorganic fertilizers increased oil yield by 39.21% compared to organic fertilizer, where O increased this characteristic by 25.5%, compared to C.

## 3. Discussion

The analysis of the results shows that the factor year did not have a statistically significant effect on the results of the study. This is a reasonable result, as there were no major differences in both temperature and rainfall between the three years. On the contrary, both plant density and fertilization had a statistically significant effect on the results. Other studies show that both density and fertilization can indeed influence the growth and yield of flax [35].

Plant height was statistically significantly influenced by fertilization. The use of urea increased this trait, because it is an easily assimilated form of nitrogen by the plant and it has been proposed that it promotes plant growth in flax [35]. On the contrary, fertilizers containing inhibitors resulted in lower plant height due to the gradual release of urea. The use of chemical fertilizers, urea and urea with inhibitors, increases plant height compared to the control [70]. Organic fertilizer also increased height, although to a lesser extent, probably due to the slow release of nutrients into the soil. According to Kakabouki et al. (2020) [71], the use of tomato pomace, either as such or in a mixture with other organic fertilizers, increased height in maize crops. Whereas in a study by Abdulazeez et al. (2025) [72], the use of organic fertilizer increased height in flax plants. As expected, the use of any fertilizer increased plant height as the concentration of nutrients in the soil increased.

The dry weight of the plant was statistically significantly affected by both factors in the experiment. Lower plant density reduced competition among plants [38], while more nutrients are provided to the plant. As a result, the plant can absorb a greater amount of nutrients which it utilizes to produce biomass [35]. In a study by Arslanoglu et al. (2022) [38] on the effect of spacing on flax cultivation, they found similar results, with density reduction increasing biomass yield. The dry weight of the plant was also affected by fertilization. The use of fertilizer increased this characteristic [73]. Increased plant biomass resulting from nitrogen (N) fertilization has been consistently reported in the literature. For instance, Soethe et al. [74] observed a positive correlation between N fertilization and flax biomass. Kakabuki et al. [35] proposed that this could be attributed to increased photosynthetic rate due to the abundance of N or the extension of the vegetative phase promoted by fertilization.

The number of capsules was statistically significantly affected by both factors of the experiment. Plant density had a statistically significant effect on the results, with low density increasing the number of capsules, probably due to greater nutrient availability to the plant per unit area [35]. Other studies suggest that the application of sparse seeding to flax crops can improve its growth [36]. Similarly, the use of fertilizer increased the number of capsules per plant. The use of inorganic fertilizers has been known to improve flax agronomic traits [75], while the use of organic fertilizers containing tomato pomace has mainly been studied in industrial tomatoes [69].

The number of seeds per capsule was not statistically significantly affected by either density or fertilization type. According to a study by Rajanna et al. (2020) [76] the number of seeds per capsule is a genetic factor, so it is reasonable that it was not affected by the factors of the experiment.

TSW was statistically significantly affected by the factors of the experiment. More specifically, the low density gave a higher TSW, probably due to the higher concentration of plant nutrients and less competition, compared to the highest density [38]. Similarly, fertilizer use helped this trait. The highest values were reported at the urea with inhibitors. This result is consistent with expectations, as there is a slow release of nitrogen into the soil, resulting in the plant being provided with nitrogen for a longer period of time [35]. Similarly, organic fertilizer increased this trait. Organic fertilizers improve some soil properties, which in turn led to better nutrient uptake by the plant [71]. Studies show that the use of organic fertilizer containing tomato pomace gave higher values in yield components [77,78].

Yield was statistically significantly affected by the interaction of density and fertilization. Higher values were recorded at higher densities with the use of inhibited fertilizer. Although in the lower densities the TSW was higher, the numerical superiority of plants per square meter, resulting from the highest density, managed to overcome this characteristic, with higher yields. At the same time, the use of inhibited fertilizer enhanced the yield of the crop. The use of inhibited fertilizers often leads to higher yields [79]. Similarly, growing plants at shorter sowing distances also leads to an increase in yields [33]. The results of this study seem to agree with those of other studies conducted with similar factors.

HI was higher under low density and the use of fertilizer with inhibitors. The wider sowing reduced the competition between plants, resulting in more nutrients being absorbed by the more sparsely sown plants. In this way, more nutrients were available for the low-density plants. In addition, the use of fertilizer with inhibitors provided crops with nitrogen for a longer period of time, avoiding its immediate absorption for stem grow. The interaction between these two factors improved HI. Both plant density [80] and fertilizers [81] have affected HI in flax crops. Angelopoulou et al. (2020) [82] reported an increase in HI of false flax, when organic fertilization was added.

Fertilization increased seed oil content. Nitrogen (N) is a crucial parameter for oil production in seeds. The use of fertilization increased this characteristic. Higher seed oil content leads to higher oil yields. As a result, oil yield was affected by the interaction between density and fertilization. According to previous studies [83,84], both seed oil content and oil yield were affected by plants density and fertilization in flax, while others reported that the use of organic fertilizers improved oil content and composition [85].

According to Table 5, agronomic characteristics are correlated with yield and yield components. The TSW correlates with plant height, dry weight and number of capsules, probably due to the efficiency of N in plants. Oil content and oil yield are influenced by all the parameters. For seed oil content, height and TSW seem to have the strongest correlation. Seed yield influences the oil yield, followed by the plants’ height and the seed oil content.

## 4. Materials and Methods

**Figure 2:**
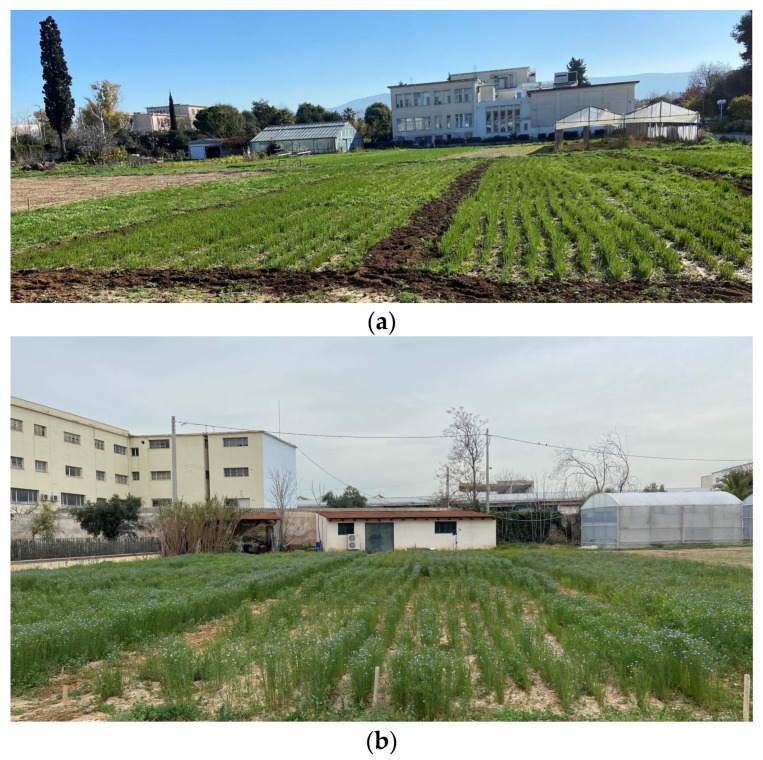
The experimental field of flax at the: (**a**) growth stage and (**b**) flowering stage.

A three-year field experiment was conducted at the Agricultural University of Athens (37°59′ N and 23°42′ E; 30 m altitude), in 2023 (I), 2024 (II) and 2025 (III) (Figure 2). The experimental area was 837 m^2^. The soil type at the experimental field was classified as clay loam, with 29.4% clay, 35.1% silt and 35.5% sand. The pH was 7.39 (1:1 H_2_O). Total nitrogen (N), phosphorus (Olsen P), potassium (K) and calcium carbonate (CaCO_3_) were measured as 0.143%, 13.6 mg kg^−1^, 233 mg kg^−1^ and 15.34%, respectively. The level of Soil Organic Matter (SOM) was 1.67%.

**Figure 3:**
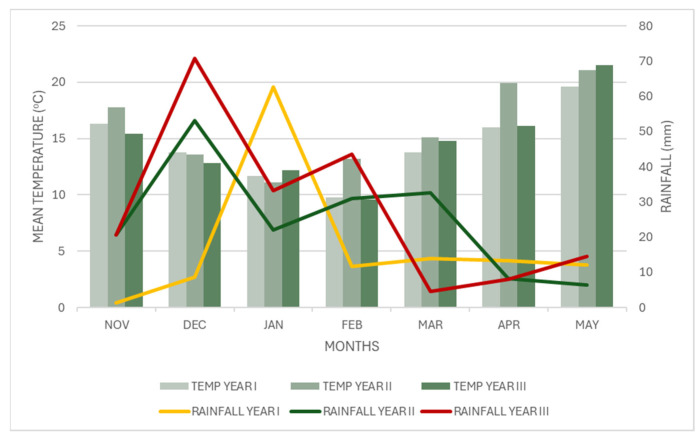
Meteorological data during the experimental period.

Data for the weather conditions were collected by an automatic weather station (Davis Vantage Pro2 Weather Station; Davis Instruments Corporation, Hayward, CA, USA), which was located in the field. Weather data collected included mean air temperature and total rainfall, during the period of the experiment. These data are presented in Figure 3. Mean temperature was 14.4, 16.0 and 14.6 °C and total rainfall was 123.7, 174.0 and 195.4 mm for years I, II and III, respectively.

The experimental design was split plot, with two factors and four replicates. Treatments were randomly assigned within each replication. In order to account for any potential field variability, replications were arranged in blocks. Each block contained all treatments. The first factor (main plot—91 m^2^) was density. Two different densities were used for the experiment. The first was 500 plants per m^2^ (D1) and the second was 300 plants per m^2^ (D2). The second factor (sub-plot—21 m^2^) was the type of fertilizer. 174 kg ha^−1^ were used for urea 46-0-0 [EUROCHEM HELLAS S.A., Athens, Greece] (U) and urea with nitrification—N-((3(5)-methyl-1H-pyrazol-1-yl) methyl) acetamide (MPA; 0.07%)- and urease—N-(2-Nitrophenyl) phosphoric triamide (2-NPT; 0.035%)- inhibitor 46-0-0 [EUROCHEM HELLAS S.A., Athens, Greece] (U + UI + NI). 2784 kg ha^−1^ of organic fertilizer was also used. The fertilization rates were selected based on the average rate proposed by the literature [10]. The composition of the organic fertilizer was tomato pomace with compost, with N total 28.7 g kg^−1^, 44% organic matter, an EC of 1.76 mS cm^−1^, a pH of 7.38, phosphorus Olsen 15 mg kg^−1^ and potassium of 30 mg kg^−1^ The last treatment was the control (C), with no fertilization.

For the three years, sowing was performed by hand on the 25th of November. Before sowing, the fertilizers were added, and they were incorporated. Flax (*Linum usitatissimum* L. cv Everest) was sown in 25 and 35 cm between rows for D1 and D2, respectively. Inside the row, the distance between the seeds was 1 cm. The crop was rainfed, so there was no irrigation. Weeds were controlled manually on a weekly basis, while infestations from pests and diseases were not observed. Harvest took place on the 9th, 10th and 10th of May for years 2023 (I), 2024 (II) and 2025 (III), respectively. Harvest was performed by hand, when the seeds’ moisture was 12%.

The measurements were performed at 160 Days After Sowing (DAS) for both years, when plants reached full maturity. These measurements referred to the estimation of agronomic characteristics. Firstly, plant height was measured. For this measurement five plants per plot were randomly chosen and clipped at ground level. After height measurement, the number of capsules per plant was measured, while the number of seeds per capsule was reported by choosing three random capsules per plant. For plant biomass, four quadrats with an area of 0.25 m^2^ each were put randomly in every plot. The plants inside the quadrat were cut at the ground level and placed in an oven, at 80 °C for 72 h. After this, matter (DM) was measured. On the harvesting day, the plants from the whole plot were collected in order to measure the Thousand Seeds Weight (TSW) and the yield. For TSW (g) one hundred seeds were randomly chosen from each treatment, four times, and weighed. The mean value of this measurement was multiplied by 10. For the yield (kg ha^−1^) measurement, seeds from every treatment were weighed and multiplied, in order to calculate the yield per ha. In order to calculate the oil content, an oil extraction from 300 g of seeds per plot was performed, via a cold pressing method. According to this method, a screw expeller SPU 20 (Senta, Serbia) was used, while the extraction took place at room temperature. Plastic bottles were used in order to store the oil samples until the following day, when they were centrifuged (3500 rpm for 15 min) and filtered through a paper filter. After that, the weight of the samples was measured, and the seed oil content was calculated by the equation: oil content (%) = (m_o_ × 100)/m_s_, where m_o_ = extracted oil’s weight (g) and m_s_ = weight of seeds’ sample (g). Oil yield was calculated by the multiplication of the yield and the oil content. Harvest Index (HI) was also measured. For the calculation of the HI was performed by the following Equation (1):

HI = (Dry weight of harvested seeds/Total dry weight of the harvested plants) × 100(1)

For the statistical analysis, the logistic package Statistica v 7.0 (Stat Soft, 2011) was used, in order to perform a two-way analysis of variance (ANOVA) for the data. Tukey’s honestly significant difference (HSD) test was used for the multiple comparison of means, while the correlation analysis was performed using Pearson’s correlation. The level of significance was 5% (*p* ≤ 0.05) for all comparisons.

## 5. Conclusions

Fertilization and plant density are two key factors significantly affecting the performance of flax in the Mediterranean region. The present study further confirms that the optimal plant density for seed oil production in flax is 300 plants m^−2^. However, the results highlight the interaction between the fertilization type and the plant density. In particular, the application of inhibited fertilizers (80 kg N ha^−1^) in combination with an average plant density of 300 plants m^−2^ can improve seed and oil yield in flax by approximately 60% and 100%, respectively, compared to the control. Lastly, the use of organic amendments that include tomato pomace showed promising results, increasing oil yield by approximately 6–50% (compared to C).

## Figures and Tables

**Table 1:** Effect of fertilization on agronomic characteristics.

	Height (cm)		Dry Weight (g plant^−1^)		Number of Capsules	
	Density					
Fertilization	D1	D2	D1	D2	D1	D2
C	56.18 ± 0.28 a	56.14 ± 0.66 a	1.14 ± 0.1 a	1.69 ± 0.13 a	13.11 ± 0.91 a	15.38 ± 1.19 a
I	64.26 ± 0.35 c	64.47 ± 0.18 c	1.38 ± 0.15 ab	2.43 ± 0.11 bc	16.95 ± 0.52 b	19.48 ± 0.33 b
O	62.61 ± 0.17 b	61.53 ± 0.37 b	1.36 ± 0.11 ab	2.1 ± 0.21 b	15.58 ± 0.32 b	17.72 ± 0.57 ab
U	64.86 ± 0.11 c	64.52 ± 0.31 c	1.48 ± 0.07 b	2.64 ± 0.08 c	16.14 ± 0.9 b	18.94 ± 1.33 b
F_YEAR_	0.01 ns		1.49 ns		2.43 ns	
F_DENSITY_	1.4 ns		99.50 ***		14.84 **	
F_FERTILIZATION_	222.13 ***		9.93 ***		7.53 **	
F_Y×D_	0 ns		2.86 ns		0.05 ns	
F_Y×F_	0.16 ns		0.94 ns		0.05 ns	
F_D×F_	1.11 ns		2.56 ns		0.05 ns	
F_Y×D×F_	0.07 ns		0.89 ns		0.04 ns	

Values represent the three-year mean (2023–2025). Different letters within the same column indicate statistically significant differences at *p* = 0.05 significance level. The symbol “ns” indicates non statistically significant differences, while the symbols “**” and “***” indicates significance at the *p* < 0.01 and *p* < 0.001 levels, respectively.

**Table 2:** Effect of plants’ density on dry weight, number of capsules and TSW.

	Dry Weight (g plant^−1^)		Number of Capsules		Thousand Seeds Weight (g)	
	Density					
Fertilization	D1	D2	D1	D2	D1	D2
C	1.14 ± 0.1 a	1.69 ± 0.13 b	13.11 ± 0.91 ns	15.38 ± 1.19ns	4.99 ± 0.14a	5.72 ± 0.31b
I	1.38 ± 0.15 a	2.43 ± 0.11 b	16.95 ± 0.52 a	19.48 ± 0.33b	7.03 ± 0.19ns	7.28 ± 0.15ns
O	1.36 ± 0.11 a	2.1 ± 0.21 b	15.58 ± 0.32 a	17.72 ± 0.57b	6.42 ± 0.37ns	6.83 ± 0.08ns
U	1.48 ± 0.07 a	2.64 ± 0.08 b	16.14 ± 0.9 ns	18.94 ± 1.33ns	6 ± 0.13ns	6.23 ± 0.08ns
F_YEAR_	1.49 ns		2.43 ns		0.76 ns	
F_DENSITY_	99.50 ***		14.84 **		6.67 *	
F_FERTILIZATION_	9.93 ***		7.53 **		23.57 ***	
F_Y×D_	2.86 ns		0.05 ns		0.04 ns	
F_Y×F_	0.94 ns		0.05 ns		0.04 ns	
F_D×F_	2.56 ns		0.05 ns		0.55 ns	
F_Y×D×F_	0.89 ns		0.04 ns		0.06 ns	

Values represent the three-year mean (2023–2025). Different letters within the same column indicate statistically significant differences at *p* = 0.05 significance level. The symbol “ns” indicates non statistically significant differences, while the symbols “*”, “**” and “***” indicates significance at the *p* < 0.05, *p* < 0.01 and *p* < 0.001 levels, respectively.

**Table 3:** Effect of fertilization on yield components.

	Seeds per Capsule		Thousand Seeds Weight (g)	
	Density			
Fertilization	D1	D2	D1	D2
C	9.29 ± 0.24 ns	9.31 ± 0.15 ns	4.99 ± 0.14 a	5.72 ± 0.31 a
I	9.78 ± 0.11 ns	9.85 ± 0.11 ns	7.03 ± 0.19 c	7.28 ± 0.15 b
O	9.38 ± 0.15 ns	9.43 ± 0.15 ns	6.42 ± 0.37 bc	6.83 ± 0.08 b
U	9.71 ± 0.17 ns	9.87 ± 0.45 ns	6 ± 0.13 b	6.23 ± 0.08 a
F_YEAR_	0.23 ns		0.76 ns	
F_DENSITY_	0.21 ns		6.67 *	
F_FERTILIZATION_	2.50 ns		23.57 ***	
F_Y×D_	0.37 ns		0.04 ns	
F_Y×F_	0.13 ns		0.04 ns	
F_D×F_	0.04 ns		0.55 ns	
F_Y×D×F_	0.24 ns		0.06 ns	

Values represent the three-year mean (2023–2025). Different letters within the same column indicate statistically significant differences at *p* = 0.05 significance level. The symbol “ns” indicates non statistically significant differences, while the symbols “*” and “***” indicates significance at the *p* < 0.05 and *p* < 0.001 levels, respectively.

**Table 4:** Effect of the type of fertilizer on seed oil content (%).

Oil Content (%)		
Density		
Fertilization	D1	D2
C	26.51 ± 1 a	27.4 ± 0.94 a
I	38.18 ± 0.71 b	39.2 ± 0.58 b
O	37.39 ± 0.29 b	38.18 ± 0.76 b
U	39.23 ± 0.95 b	38.71 ± 0.64 b
F_YEAR_	0.12 ns	
F_DENSITY_	0.87 ns	
F_FERTILIZATION_	97.08 ***	
F_Y×D_	0.10 ns	
F_Y×F_	0.31 ns	
F_D×F_	0.37 ns	
F_Y×D×F_	0.11 ns	

Values represent the three-year mean (2023–2025). Different letters within the same column indicate statistically significant differences at *p* = 0.05 significance level. The symbol “ns” indicates non statistically significant differences, while the symbols “***” indicates significance at the *p* < 0.001 levels.

**Table 5:** Correlation coefficients between the agronomic characteristics, the yield and the yield components of flax crop.

	Height	Dry Weight	Number of Capsules	Seeds per Capsule	TSW	Yield	HI	Oil Content	Oil Yield
Height		0.30 **	0.39 ***	0.27 **	0.52 ***	0.37 ***	0.37 ***	0.82 ***	0.75 ***
Dry weight			0.39 ***	0.12 ns	0.33 ***	0.02 ns	0.35 ***	0.34 ***	0.23 *
Number of capsules				0.04 ns	0.31 **	0.10 ns	0.35 ***	0.40 ***	0.33 **
Seeds per capsule					0.15 ns	0.16 ns	0.02 ns	0.24 *	0.27 *
TSW						0.19 ns	0.45 ***	0.58 ***	0.49 ***
Yield							0.27 **	0.19 *	0.81 ***
HI								0.36 ***	0.41 ***
Oil content									0.72 ***
Oil yield									

Different letters within the same column indicate statistically significant differences at *p* = 0.05 significance level. The symbol “ns” indicates non statistically significant differences, while the symbols “*”, “**” and “***” indicates significance at the *p* < 0.05, *p* < 0.01 and *p* < 0.001 levels, respectively.

## Data Availability

All data supporting the reported results are provided in the tables within the text.

## Abbreviations

The following abbreviations are used in this manuscript:

ACs	Alternative crops
ALA	Alpha-linolenic acid
N	Nitrogen
NBPT	Thiophosphoricriamide
DCD	Dicyandiamide
PGPM	Plant growth promoting microorganisms
TSW	Thousand seeds weight
NI	Nitrification inhibitor
UI	Urease inhibitor
D	Density
HI	Harvest index
P	Phosphorus
K	Potassium
CaCO_3_	Calcium carbonate
SOM	Soil organic matter
